# Semaphorin 4D Induces an Imbalance of Th17/Treg Cells by Activating the Aryl Hydrocarbon Receptor in Ankylosing Spondylitis

**DOI:** 10.3389/fimmu.2020.02151

**Published:** 2020-09-08

**Authors:** Jianmin Xie, Zitao Wang, Wen Wang

**Affiliations:** Department of Rheumatology, The Second Affiliated Hospital of Nanjing Medical University, Nanjing, China

**Keywords:** ankylosing spondylitis, semaphorin 4D, Th17, Treg, aryl hydrocarbon receptors

## Abstract

**Objectives:**

Semaphorin 4D (Sema4D) is constitutively expressed on T cells and osteoclasts, and regulates T cell proliferation and bone remodeling. In addition, several studies have shown that Sema4D is involved in the pathogenesis of autoimmunity. We undertook this study to investigate the mechanism by which Sema4D affects the pathogenic progress of ankylosing spondylitis (AS).

**Methods:**

Soluble Sema4D (sSema4D) levels in serum were analyzed by enzyme-linked immunosorbent assay. The cell surface levels and transcripts of Sema4D were evaluated in CD4 + and CD19 + cells from the AS patients and healthy individuals. The mRNA expression levels were assessed by quantitative polymerase chain reaction (qPCR). The proportions of Treg cells and IL-17-producing T-cells (Th17 cells) differentiated from CD4 + T cells were analyzed by flow cytometric analysis. The aryl hydrocarbon receptor (AhR) agonistic effect of Sema4D was detected by analyzing the activation of downstream signaling pathways and target genes using Luciferase and EROD assay.

**Results:**

Levels of sSema4D were elevated in both serum from AS patients, and clinical features markers were correlated with serum sSema4D levels. Sema4D facilitated CD4 + T cells proliferation and Th17 cells differentiation and inhibited Treg cells differentiation by enhancing RORγt expression and reducing Foxp3 expression, with increasing expression and secretion of IL-17 and IL-22. It induced the expression and activity of AhR target gene CYP1A1 and XRE reporter activity *via* interaction with CD72.

**Conclusion:**

These findings indicate that Sema4D as a potent activator of T cells in the immune response contributes to the inflammation of AS by inducing imbalance in Th17 and Treg cell populations in an AhR-dependent manner, suggesting it is a crucial participant in AS pathogenesis.

## Introduction

Ankylosing spondylitis (AS) is a chronic autoinflammatory rheumatic disease characterized by immunoinflammatory responses and abnormal bone remodeling. Features such as inflammatory cytokine production, syndesmophytes, erosions and osteoporosis are manifestations of progressive AS (1, 2). Accumulating findings have demonstrated that T helper 17 (Th17) cells, a subgroup of CD4 + T cells, secrete interleukin (IL)-17, enhance proinflammatory effects and aggravate AS. Conversely, regulatory T (Treg) cells contribute critically to protection against pathogenic T cell responses and the maintenance of dominant immune homeostasis (3). Recent studies indicate that AS is significantly associated with the number of peripheral blood Th17 cells (4). Increasing numbers of reports have verified that the ratio of Th17 to Treg cells is elevated in AS patients, suggesting that the balance between Th17 and Treg cells may be a crucial factor for AS development (4, 5). In addition, inflammatory cytokines, immune cells and osteocytes might be responsible for bone remodeling in AS. The pathophysiology of AS is one of abnormal bone metabolism characterized by pathological loss of trabecular bone in the center of the vertebral bodies accompanied by new bone formation in the cortical regions of the vertebrae (6, 7). To date, the exact pathogenesis of immune activation and abnormal bone remodeling in AS remains obscure. To allow AS patients to attain true disease remission, seeking another pivotal molecular player that promotes immune activation and bone loss in AS is an indispensable task.

Accumulating evidence has showed that the aryl hydrocarbon receptor (AhR) (8, 9), a ligand-dependent transcriptional factor, is crucial for eliciting the responses and developing the immune function of Th17 and Treg cells, performing a particularly critical function in autoimmune diseases such as rheumatoid arthritis (RA). Recent studies conducted by experts on AhR have shown the function of AhR in regulating Th17 and Treg cell differentiation. According to the type of ligand, activation of AhR can induce Th17 or Treg cell differentiation, leading to aggravation of proinflammatory or immunosuppressive responses, respectively, in autoimmune diseases (10–12). AhR activation by its high-affinity ligand 6 formylindolo[3,2-b]carbazole (FICZ) may induce Th17 cells and promote IL-17 production (13). However, the effect of AHR on Th17 and Treg cell differentiation in AS has not been well studied.

The semaphorins are a family of more than 20 proteins that are implicated in cell-to-cell communication and can be divided into eight main classes (14). Notably, recent research on semaphorins demonstrated that these proteins are critical for the immune response and bone metabolism (15, 16). In the central nervous system, semaphorin 4D (Sema4D) was originally identified as an axon guidance molecule, but in the immune system, it was originally identified as a T cell activation marker that is constitutively expressed on T cells and regulates T cell priming (17, 18). Sema4D has been shown to be implicated in the development of rheumatoid arthritis (19). Interestingly, Sema4D was recently identified in the bone microenvironment and demonstrated to be a product of osteoclasts that acts on osteoblasts to inhibit bone formation, thereby disrupting bone homeostasis in favor of resorption. Knockdown of Sema4D prevents bone loss, suggesting that Sema4D may be a new and potentially effective target for drugs that promote bone formation (20, 21). Immune and bone metabolism abnormalities have critical roles in the progression of AS, suggesting that Sema4D might exacerbate AS. However, the involvement of Sema4D in the pathogenesis of AS has not yet been verified. Based on the previously reported role of Sema4D, we hypothesized that Sema4D may be involved in regulating Th17 and Treg cell differentiation through AhR activation, causing an inflammatory response and inducing the bone remodeling process in AS patients.

Although extensive studies have addressed the physiological and pathological effects of Sema4D on many autoimmune diseases, the potential role of Sema4D in immunoregulation and bone remodeling in AS pathogenesis has not yet been reported. Thus, the present study was conducted to clarify the potential role of Sema4D in AS patients and to explore the underlying mechanism by which Sema4D could enhance the differentiation of proinflammatory Th17 cells and suppress the differentiation of anti-inflammatory Treg cells through activation of AhR, which requires the Sema4D-CD72 interaction.

## Subjects and Methods

### Study Subjects

Serum samples from 56 patients with AS and 43 age- and sex-matched healthy controls were collected with informed consent in accordance with the principles of the Declaration of Helsinki for research involving human subjects and with approval from the local ethics committee of Nanjing Medical University. The AS patients fulfilled the 1984 modified New York criteria for the diagnosis of AS and were naïve to systemic treatment (22, 23). No subject had other systemic diseases, autoimmune diseases, active infectious processes, a known history of bone fractures in the previous 24 months, a history of neurological cognitive disease, or a history of osteoporosis. To compare sema4D expression levels before and after anti-TNF-α treatment, we monitored the expression of sema4D in 20 patients who were treated with a TNF-α blocker after enrollment in the present study. Patient serum specimens were stored at −80°C until analysis as described below. PBMCs from healthy donors and patients with AS were isolated by discontinuous density gradient centrifugation, washed twice in sterile phosphate-buffered saline (PBS) and resuspended at a concentration of 1 × 10^6^ cells/ml PBS.

### Clinical and Laboratory Assessment

We recorded the disease duration, BMI, sex, age, and extraarticular manifestations of AS patients. The disease activity of AS was assessed by the CRP level and Bath AS disease activity index (BASDAI). BAP (San Diego, CA, United States), osteocalcin (Nordic Biosciences, Herlev, Denmark), TRACP-5b and C-terminal cross-linking telopeptide of type I collagen (CTX) (Nordic Biosciences) were evaluated as markers of bone turnover. Serum BAP and TRACP-5b levels were measured by enzyme-linked immunosorbent assay (ELISA). Osteocalcin and CTX levels were measured by electrochemiluminescence immunoassays (ECLIAs) (24). The intraassay and interassay coefficients of variation were less than 9% for all parameters. The assay was performed according to the manufacturer’s instructions. Complete blood counts were obtained, routine biochemical analyses were conducted, and HLA-B27 was evaluated.

### Serum Sema4D Levels and Cytokine Analysis

The serum concentrations of Sema4D were determined using commercially available ELISA kits (MyBio-Source, San Diego, CA, United States). Assessment was performed according to the manufacturer’s instructions. The levels of soluble IL-17, IL-22 and IL-10 were measured using ELISA kits specific for each cytokine (R&D Systems, Minneapolis, MN, United States). All measurements were performed in triplicate.

### CD4 + T Cell Purification and Stimulation

PBMCs were isolated from blood samples freshly obtained from AS patients and healthy controls in lymphocyte separation medium (San Diego, CA, United States) according to the manufacturer’s instructions. Isolation of CD4^+^ T cells from PBMCs was performed by magnetic cell separation. MACS CD4 microbeads (Miltenyi Biotec, Auburn, CA, United States) were incubated with PBMCs and applied to a MidiMACS separation column (Miltenyi Biotec, Auburn, CA, United States) according to the manufacturer’s instructions.

The purity of the isolated CD4^+^ T cells was determined by flow cytometry to be >95% for each population. The purified CD4 + T cells were seeded in 96-well plates at 1 × 10^6^ cells per well and were then stimulated with or without soluble human Sema4D-Fc fusion protein (PeproTech, Rocky Hill, NJ, United States), anti-Plexin B2 (PLXNB2) antibody, anti-Plexin B1 (PLXNB1) antibody or anti-CD72 ligation antibody (BU40; Santa Cruz Biotechnology, United States). To assay Th17 cell differentiation *in vitro* (25), cells were induced to differentiate into Th17 cells with anti-CD3 (2 μg/ml, plate-bound) and anti-CD28 (2 μg/ml, soluble) antibodies. For blocking assays, cells were cocultured with 10 ng/ml Sema4D-Fc and 10 ng/ml anti-Sema4D antibody or isotype-matched control IgG for 48 h. The concentrations of human IL-10, IL-22 and IL-17 in culture supernatants were determined by ELISA. At the end of the stimulation period, cells were collected and analyzed by flow cytometry. Quantitative RT-PCR analysis (qRT-PCR) was performed as described above.

### Flow Cytometric Analysis

CD4 + T cells were harvested before and after stimulation. Cell surface markers were stained with the indicated labeled antibodies against the indicated cell surface antigens. Cells were prepared in heparinized tubes by Ficoll-Paque density gradient centrifugation and were then analyzed on a FACSCanto (Invitrogen, Carlsbad, CA, United States) using FlowJo software (Tree Star) according to the manufacturer’s instructions. The following antibodies were used for flow cytometry to analyze the cell types and cytokine production: PE-CD4, Foxp3-APC, CD25-PE and FITC-IL-17A (BioLegend, CA, United States). FITC-, PE- and APC labeled mouse IgG antibodies were utilized as isotype controls (BioLegend, CA, United States).

### Proliferation Assay

For the proliferation assay, isolated CD4^+^ T cells were labeled with a Cell TraceTM CFSE Cell Proliferation Kit (Invitrogen, Carlsbad, CA, United States) at a final concentration of 4 μM. CFSE-labeled CD4^+^ T cells were incubated under the described conditions. A total of 1 × 10^6^ CFSE-labeled T cells were seeded into a flat-bottom 96-well plate. Soluble anti-sema4D (see above), soluble anti-CD72 (BioLegend, San Diego, CA, United States), or matched isotype antibodies were added as indicated. T cell proliferation was recorded after 3 and 5 days based on CFSE dilution as measured using flow cytometry.

### Western Blot Assay

Cells were collected after induction, and cell lysate was prepared from 1 × 10^7^ cells. The Western blot assay was performed according to the manufacturer’s protocols.

### RNA Extraction and qRT-PCR

To measure the mRNA expression levels of IL-17A, ROR-γt, Foxp3, and GAPDH, total RNA from human PBMCs and CD4 + T cells was extracted using a QIAGEN RNeasy Mini Kit (QIAGEN, Hilden, Germany), and complementary DNA (cDNA) was synthesized using a SuperScript II cDNA Synthesis Kit (Invitrogen, Carlsbad, CA, United States) according to the manufacturer’s protocols. The primer sequences were as follows: IL-17, forward, 5′-CGGACTGTGATGGTCAACCTGA-3′,reverse,5′-GCACTTT GCCTCCCAGATCACA-3′; FoxP3,forward,5′-GGCACAATG TCTCCTCCAGAGA-3′,reverse,5′-CAGATGAAGCCTTGGTC AGTGC-3′;ROR-γt,forward,5′-CAGAATGACCA-GATTGTGC TT-3′,reverse,5′-TCCATGCCACCGTATTTGC-3′;AhR,forward, 5′-CAAATCAGAGACTGGCAGGA-3′,reverse,5′-AGAAGACC AAGGCATCTGCT-3′;CYP1A1,forward,5′-GTTCTTGGAGCT TCCCCGAT-3′,reverse,5′-CTGACACGAAGGCTGGAAGT-3′, and GAPDH,forward,5′-GTCTCCTCTGACTTCAACAGCG-3′, reverse,5′-ACCACCCTGTTGCTGTAGCCAA-3′. All reactions were carried out in triplicate in the same plate.

### Transfection

CD4^+^ T cells were transfected with siAhR for 24 h using Lipofectamine 2000 according to the manufacturer’s protocols. SiGENOME RISC-free Control siRNA was used as the control. The cells were then rinsed, and then exposed to 10 ng/ml Sema4D in fresh media for 24 h (26).

### Cell Culture and Luciferase Assay

EL-4 cells were cultured at 37°C in an atmosphere containing 5% CO2 in RPMI 1640 medium (Gibco, United States) supplemented with 10% heat-inactivated fetal bovine serum. EL-4 cells were plated in 96-well plates (1 × 10^6^ cells per well), and the cells in each well were cotransfected with the pGL3 [luc2P/XRE/Hygro] vector containing a xenobiotic response element (XRE) that drives the transcription of the luciferase reporter gene luc 2P (*Photinus pyralis*) (27). Fresh culture medium was added to the cells along with FICZ (300 nM), Sema4D (10 ng/ml), anti-PlexinB1 antibody (5 μg/ml), anti-PlexinB2 antibody (5 μg/ml), anti-CD72 antibody (5 μg/ml) or CH223191 (30 μM) either alone or in combination. The CD4 + T cells and EL-4 cells were incubated for 12 h, and luciferase activity was then measured with a luciferase assay system (Promega, Madison, WI, United States) in a multimode reader according to the manufacturer’s instructions.

### Ethoxyresorufin-O-Deethylase (EROD) Activity

EL-4 cells were plated into 6-well plates at a density of 1 × 10^6^ cells/ml; stimulated with Sema4D, anti-PlexinB1 antibody, anti-PlexinB2 antibody, anti-CD72, CH223191, and FICZ either alone or in combination; and incubated for 24 h. The supernatant was then collected. Then, CYP1A1 activity was measured with an EROD enzyme assay, as previously described (28). Fluorescence intensity was detected by using a FL600 plate reader (Biotek, Winooski, VT, United States), with excitation at 530 nm and emission at 590 nm.

### Statistical Analysis

Statistical significance was calculated using SPSS 20.0. The non-parametric Mann-Whitney *U* test was used for comparisons between 2 groups, and comparisons among 3 groups were performed using the Kruskal-Wallis test followed by the Mann-Whitney *U* test. Correlation analysis was performed using the Pearson correlation test. For all statistical analyses, *p* values of less than 0.05 were considered significant.

## Results

### Sema4D Levels Were Significantly Elevated in Patients With AS

**FIGURE 1:**
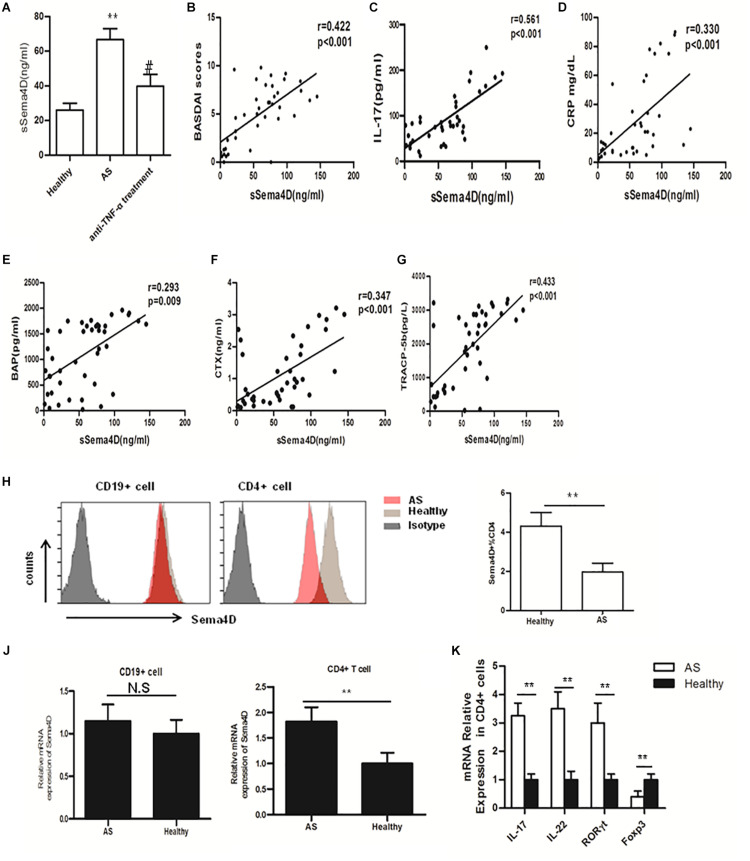
Sema4D and cytokine expression, and correlations of serum levels of soluble Sema4D with clinical features. **(A)** Serum sSema4D levels before and after anti-TNF-α treatment in 20 good responders according to the European League Against Rheumatism response criteria. ** = *p* < 0.001 vs Healthy; # = *p* < 0.01 vs AS. **(B–D)** Positive correlation of serum sSema4D levels with the Bath AS disease activity index (BASDAI), the level of IL-17, and the C-reactive protein (CRP) level. **(E–G)** correlation of serum sSema4D levels with serum bone remodeling marker levels in AS patients. **(H)** Cell surface expression of Sema4D in peripheral blood CD4+, and CD19 + cells. Results shown are representative of findings from 6 Ankylosing Spondylitis (AS) patients and 6 healthy controls. **(J)** mRNA expression of Sema4D in peripheral blood CD4 + and CD19 + cells. Results shown are from 20 AS patients and 15 healthy controls. **(K)** mRNA expression of IL-17, IL-22, RORγt and Foxp3 in peripheral blood CD4 + cells. Results shown are from 20 AS patients and 15 healthy controls. The data were presented as means ± SEM. The results are representative of three independent experiments ** = *p* < 0.01.

Recently, the effects of sema4D on the immune system have been shown to play critical roles in diverse pathological processes in many chronic inflammatory diseases, such as RA. However, the specific role of Sema4D in modulating immune inflammation in AS has not yet been elucidated. To investigate the pathologic implications of Sema4D in patients with AS, we measured serum concentrations of Sema4D in AS patients and healthy controls. As shown in Figure 1A, serum concentrations of soluble Sema4D were significantly higher in AS patients than in healthy controls (mean ± SD 66.7 ± 6.9 ng/ml vs 26.8 ± 3.9 ng/ml; *p* < 0.01). Importantly, serum Sema4D levels were significantly decreased after 2 months of treatment with a TNF-α blocker. Previous reports showed that the levels of IL-17 in AS patients were significantly higher but those of sclerostin were significantly lower than the corresponding levels in controls. The present results are consistent with the previous reports. The clinical data and baseline demographic informations of participants are presented in Table 1. Serum concentrations of soluble Sema4D were significantly correlated with Bath AS disease activity index (BASDAI), CRP and serum IL-17 levels (*r* = 0.422 and *p* < 0.001 for BASDAI; *r* = 0.330 and *p* < 0.001 for CRP; *r* = 0.561 and *p* < 0.001 for IL-17) (Figures 1B–D). However, no significant correlation was found between the levels of soluble Sema4D and sclerostin (not shown).

**TABLE 1:** Baseline characteristics of study population.

Characteristics	AS (*n* = 56)	HC (*n* = 43)	*p* value
Age (yrs old)	35.24 ± 13.37	32.36 ± 12.37	NS
Male/female	42/14	30/13	NS
Disease duration (yr)	14.2 ± 9.7		
Axial with peripheral arthritis/axial			
Disease only (patient number)	33/23		
HLA-B27 (+) (%)	52 (92.8%)		
CRP (mg/dl)	23.2 ± 12.5		
ESR (mm/h)	24.9 ± 15.3		
BASDAI	5.2 ± 3.4		
Schober’s test (cm)	3.56 ± 2.18		
Finger to floor (cm)	17.8 ± 14.9		
Chest expansion (cm)	4.47 ± 1.56		
Right lateral bending (cm)	8.32 ± 4.26		
Left lateral bending (cm)	8.58 ± 7.13		
Occipital to wall (cm)	4.23 ± 6.57		
Tragus to wall (cm)	12.5 ± 6.24		
Intramalleolar distance (cm)	93.22 ± 20.15		
Cervical spine lateral rotation,	43.4 ± 29.8		
right (degree)			
Cervical spine lateral rotation,	46.8 ± 27.3		
left (degree)			

*AS, Ankylosing spondylitis; HC, healthy control; NS, non-significant; HLA-B27, human leukocyte antigen-B27; CRP, Creactive protein; ESR, erythrocyte sedimentation rate; BASDAI, Bath Ankylosing spondylitis Disease Activity Index; yr, year. Data are presented as mean standard deviation.*

### Correlation Between the Serum Sema4D Levels and Serum Bone Remodeling Marker Levels

Accumulating data have identified Sema4D as a product of osteoclasts, which skew bone homeostasis toward resorption and are involved in the bone remodeling process (21, 29). To determine whether the serum Sema4D concentration is correlated with serum bone remodeling marker levels in AS patients, we measured the serum levels of bone remodeling markers. The serum levels of BAP, TRAP 5b, CTX-I and OC in AS patients were significantly elevated compared with those in healthy controls (1123 ± 98.7 vs 877.9 ± 49.5 pg/ml, *p* < 0.001; 1869 ± 169.3 vs 1672 ± 112.8 pg/L, *p* < 0.001; 1.92 ± 0.45 vs 1.2 ± 0.23 ng/ml, *p* < 0.01 and 14.2 ± 4.7 vs 13.1 ± 5.3, *p* = 0.057, respectively). More importantly, significant positive correlations were found between the serum Sema4D concentration and serum levels of the bone turnover markers CTX-I, TRAP 5b and BAP (*r* = 0.347, *p* < 0.001; *r* = 0.433, *p* < 0.001; *r* = 0.293, *p* = 0.009, respectively) (Figures 1E–G), whereas no correlations were found between the serum Sema4D concentration and serum levels of the bone formation marker OCN. Additionally, no significant correlations were found between the serum Sema4D concentration and age or BMI. These data suggested that Sema4D plays a critical role in bone resorption and might be involved in the pathogenesis of bone loss in AS patients.

### Sema4D Expression in the CD4^+^ T Cells of AS Patients

Sema4D has been reported to exhibit higher expression levels on T cells than on other lymphocytes, such as B cells, in RA patients. In addition, Sema4D expression is further enhanced after cellular activation (30, 31). However, the role of Sema4D in the pathogenesis of AS has not been defined. PBMCs were isolated from AS patients and healthy controls, and Sema4D expression was detected in PBMCs from AS patients and healthy controls by flow cytometry. In healthy controls, cell surface membrane-bound Sema4D was abundantly expressed on CD4 + T cells but was expressed at lower levels on CD19 + B lymphocytes. By contrast, cell surface expression of Sema4D was significantly downregulated on CD4 + T cells from AS patients compared with those from healthy donors (Figure 1H). Interestingly, qRT-PCR revealed that the mRNA expression of Sema4D in CD4 + T cells was elevated in AS patients (Figure 1J), suggesting that the increased serum levels of soluble Sema4D and the reduced levels of cell surface membrane-bound Sema4D on CD4 + T cells in patients with AS were due to shedding of Sema4D from the surface of activated T cells. As Sema4D expression was significantly enhanced in CD4 + T cells isolated from PBMCs of AS patients, we then analyzed the expression levels of other cytokines and transcription factors in CD4 + T cells isolated from PBMCs of AS patients. Our data revealed that compared with those of healthy controls, CD4 + T cells isolated from PBMCs of AS patients exhibited increased levels of IL-17A and RORγt mRNA but a decreased level of Foxp3 mRNA (Figure 1K).

### Sema4D Promotes CD4 + T Cell Proliferation and Th17 Cell Differentiation, Whereas Inhibits Treg Cell Differentiation

**FIGURE 2:**
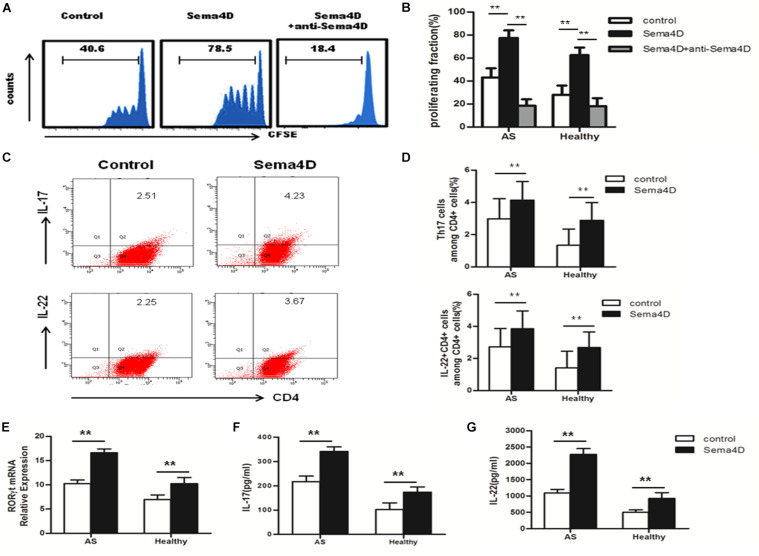
Sema4D promoted CD4 + T cell proliferation and Th17 cell differentiation. **(A,B)** CFSE labeled CD4 + T cells from AS patients (*n* = 20) and Healthy controls (*n* = 15) were treated with Sema4D (10 ng/ml) or anti-Sema4D antibody (10 ng/ml) for 3 days. **(A)** Showed a representative result of flow cytometry of AS patients. **(C,D)** The proportion of Th17 cells (CD4 + IL-17 + cells) and CD4 + IL-22 + cells in CD4 + T cells of AS patients (*n* = 20) and Healthy controls (*n* = 15) treated with or without Seam4D (10 ng/ml) for 3 days. **(C)** Showed a representative result of FACS of AS patients. **(E)** RORγt mRNA expression in CD4 + T cells from AS patients (*n* = 20) and Healthy controls (n = 15) treated with or without Sema4D (10ng/ml). **(F,G)** Sema4D induced Th17-related cytokines production. IL-17 and IL-22 levels in the supernatant of CD4 + T cells from AS patients (*n* = 20) and Healthy controls (*n* = 15) treated with or without Sema4D (10 ng/ml) for 3 days by ELISA. The data were presented as means ± SEM. The results are representative of three independent experiments ** = *p* < 0.01.

Previous reports have suggested that soluble Sema4D exerts multiple effects on CD4 + T cells in several diseases (10, 11). Thus, we speculated that Sema4D may also affect CD4 + T cells in AS patients. CD4 + T cells were harvested from PBMCs of AS patients and healthy donors, stained with CFSE, and cocultured with or without soluble human Sema4D-Fc in the presence of an anti-Sema4D or isotype control antibody. After 3 days, the proliferation rates and proportions of T cell subsets were assessed by flow cytometry. Notably, after stimulation with Sema4D, CD4 + T cells from AS patients showed significant proliferation (Figures 2A,B), which was markedly suppressed by adding soluble anti-Sema4D.

**FIGURE 3:**
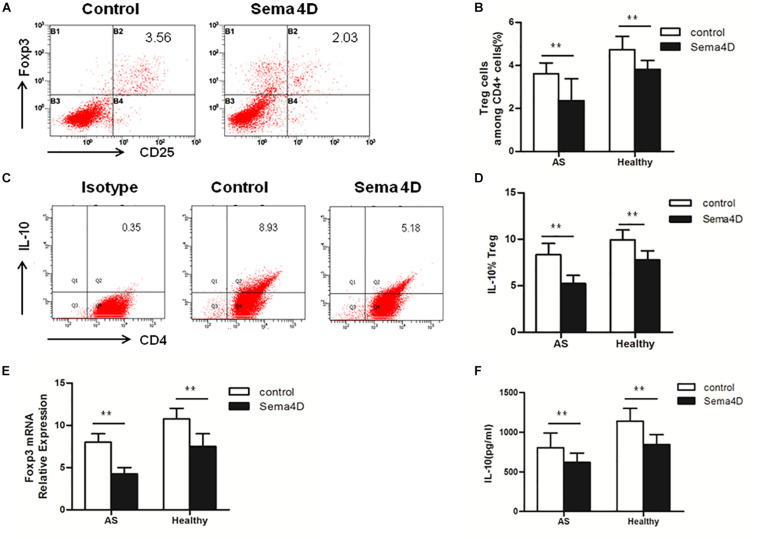
Sema4D (10 ng/ml) inhibited Treg cell differentiation *in vitro*. **(A,B)** The proportion of Treg cells (CD4+, CD25+, and Foxp3 + cells) in CD4 + T cells from AS patients (*n* = 20) and Healthy controls (*n* = 15) treated with or without Sema4D (10 ng/ml) for 3 days. **(A)** Showed a representative FACS result of AS patients. **(C,D)** The expression of IL-10 of Treg cells in CD4 + T cells of AS patients (*n* = 20) and Healthy controls (*n* = 15) treated with or without Sema4D (10 ng/ml) for 3 days. **(C)** Showed a representative FACS result of AS patients. **(E)** Foxp3 mRNA expression in CD4 + T cells from AS patients (*n* = 20) and Healthy controls (*n* = 15) treated with or without Sema4D (10 ng/ml). **(F)** IL-10 was quantified in the supernatant of CD4 + T cells from Healthy (*n* = 15) and AS patients (*n* = 20) treated with or without Sema4D (10 ng/ml) for 3 days by CBA. The data were presented as means ± SEM. The results are representative of three independent experiments, ** = *p* < 0.01.

**FIGURE 4:**
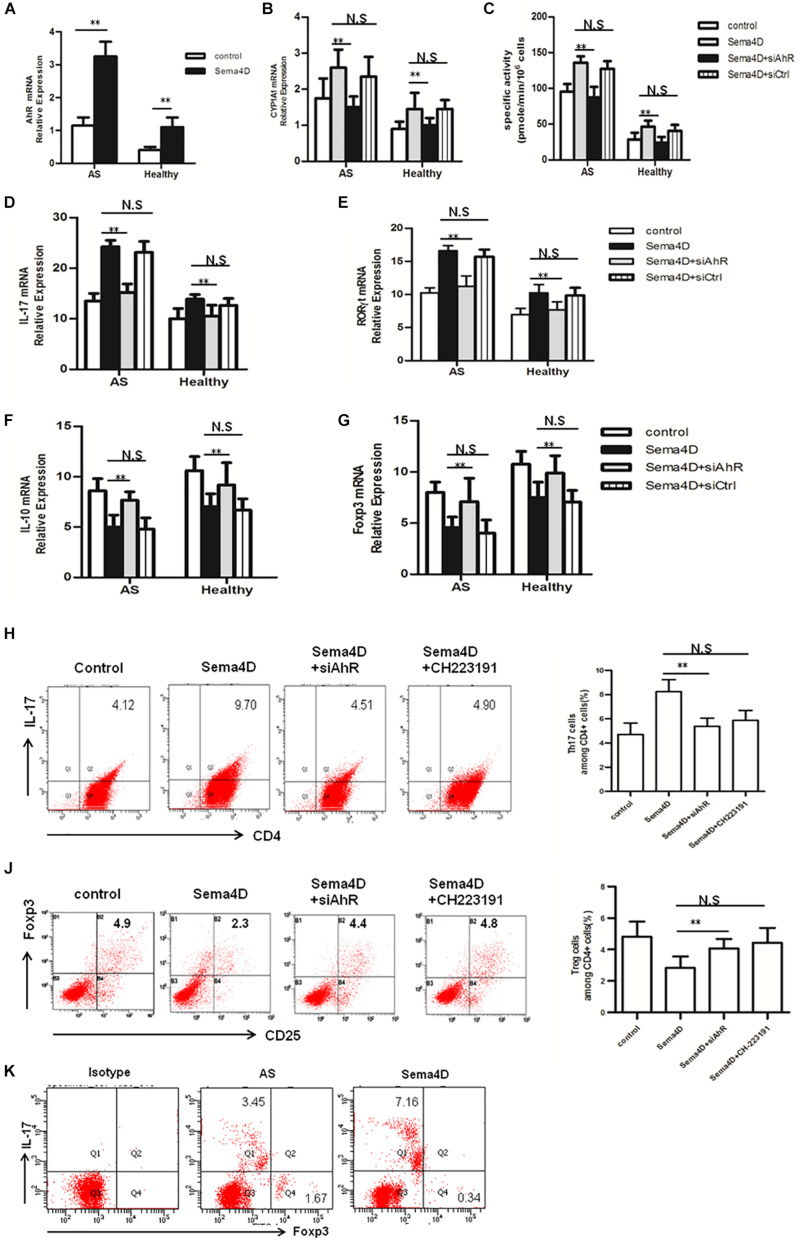
AhR antagonist and siAhR abolished Sema4D-mediated regulation in Th17/Treg differentiation *in vitro*. **(A)** The mRNA levels of AhR were analyzed in CD4 + T cells from AS patients (*n* = 20) and Healthy controls (*n* = 15) treated with or without Sema4D (10 ng/ml) using a quantitative PCR assay. **(B,C)** Enzymatic activity and mRNA levels of CYP1A1 in CD4 + T cells from AS patients (*n* = 20) and Healthy controls (*n* = 15) treated with Sema4D (10 ng/ml) or siAhR plasmid were evaluated by EROD and qRT-PCR, respectively. **(D–G)** mRNA levels of IL-17, RORγt, IL-10 and Foxp3 in CD4 + T cells from AS patients (*n* = 20) and Healthy controls (*n* = 15) treated with Sema4D (10 ng/ml) or siAhR plasmid were evaluated by qRT-PCR, respectively. **(H–J)** CD4 + cells of AS patients were stained with antibodies against CD4/IL-17 and CD4/CD25/Foxp3. Frequency of IL-17A + cells in CD4 + cells and frequency of CD25 + Foxp3 + cells in CD4 + cells treated with Sema4D (10 ng/ml), CH223191 (30 μM) or siAhR plasmid were analyzed by flow cytometry. The data were presented as means ± SEM. **(K)** Flow cytometry analysis was performed on CD4 + T cells treated with Sema4D to identify Foxp3 + Treg cells and IL-17 + Th17 cells. The results are representative of three independent experiments. ** = *p* < 0.01. N.S = no significant.

To further explore the effect of Sema4D on CD4 + T cells subsets, Th17 and Treg cell differentiation, we used qRT-PCR to measure the mRNA expression levels of transcription factors. The expression of RORγt, a transcription factor specifically promoting Th17 cell differentiation, was significantly increased in CD4 + T cells from both AS patients and healthy controls after stimulation with Sema4D (Figure 2E). Notably, we found that the expression of Foxp3, which is critical for the function and differentiation of Treg cells, was decreased in CD4 + T cells from both AS patients and healthy controls after stimulation with Sema4D (Figure 3E). Taken together, these data implied that soluble Sema4D alone can directly activate CD4 + T cell proliferation and Th17 cell differentiation even in the absence of other stimulatory cytokines. Due to the increased expression of RORγt and decreased expression of Foxp3, we next investigated whether soluble Sema4D increases Th17 cell numbers and decreases Treg cell numbers. We used flow cytometry to measure the proportions of Treg cells and Th17 cells and found that the proportion of Th17 cells was increased and that of Treg cells was decreased among the CD4 + T cell populations from both healthy controls and AS patients after Sema4D stimulation (Figures 2C,D, 3A,B). Furthermore, we measured IL-17 and Foxp3 expression in the CD4 + T cells from AS patients after stimulation with Sema4D, and found that Sema4D enhanced IL-17 expression and reduced Foxp3 expression (Figure 4K).

Subsequently, we examined the protein levels of molecules implicated in Th17 cell differentiation (IL-22 and IL-17) and Treg cell differentiation (IL-10) in CD4 + T cells from AS patients and healthy controls following treatment with Sema4D. Sema4D significantly elevated the protein levels of IL-17 and IL-22 (Figures 2F,G), and reduced the protein levels of the IL-10 (Figures 3C,D,F).

### The AhR Pathway Is Involved in Sema4D-Mediated Th17 Cell and Treg Cell Differentiation

Recently, an increasing number of reports have elicited that AhR playes a critical role in the regulation of Th17 and Treg cell differentiation (12, 32). Thus, we investigated whether AhR contributes to the Sema4D-mediated the balance of Th17/Treg cells, CD4 + T cells were transfected with siAhR in the presence of Sema4D under Th17- or Treg-polarizing conditions, and the expression levels of AhR and the AhR target gene CYP1A1 were measured. Of note, Sema4D could enhance CYP1A1 activity and AhR expression under Th17 or Treg-polarizing conditions. Interestingly, knockdown of AhR reduces CYP1A1 activity and expression (Figures 4A–C). Next, We found that AhR knockdown markedly diminished the enhanced effect of Sema4D on the expression of the IL-17 and RORγt mRNA (Figures 4D,E), and increased the IL-10 and Foxp3 mRNA expression (Figures 4F,G). Furthermore, our results showed that the proportion of Th17 cells was reduced and those of Treg cells was elevated in the CD4 + T cells from healthy individuals and AS patients after stimulation with Sema4D and siAhR plasmid comparing to stimulation with Sema4D (Figures 4H,J). All these data suggested that Sema4D-promoted expression of CYP1A1 might be dependent on AhR pathway activation.

**FIGURE 5:**
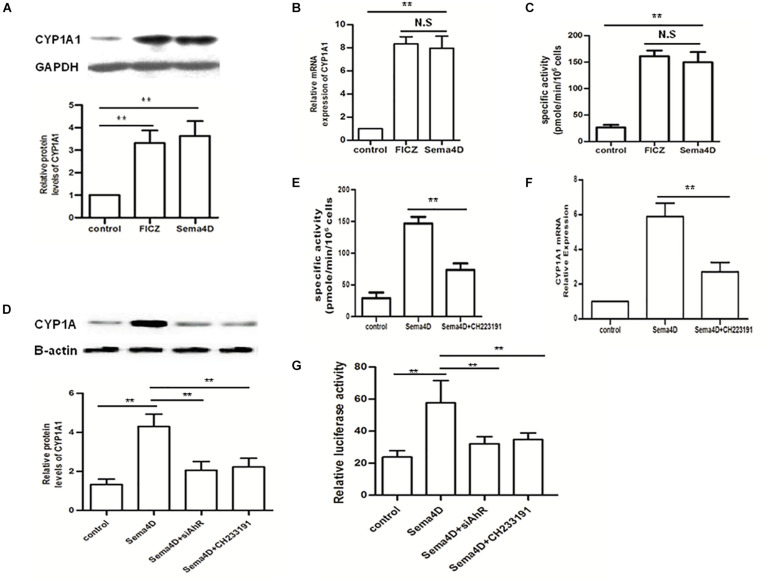
Effect of Sema4D on CYP1A1 activity in the EL-4 cells. **(A–C)** Protein, mRNA and enzymatic activity levels of CYP1A1 were analyzed using Western blot, qRT-PCR and EROD assay, respectively after 12 h treatment with FICZ (300 nM) or Sema4D (10 ng/ml). **(D–F)** Protein, enzymatic activity and mRNA levels of CYP1A1 were analyzed using Western blot, qPCR and EROD assay, respectively after 12 h treatment with Sema4D (10 ng/ml), CH233191 (30 μM) or siAhR plasmid. **(G)** EL-4 cells were transfected with the XRE–Luc construct and then subjected to indicated treatments for 12 h. Sema4D-induced XRE promoter activity was assayed by luciferase activity. The data were presented as means ± SEM. The results are representative of three independent experiments, ** = *p* < 0.01; N.S = no significant.

To further evaluate effect of Sema4D on the AhR pathway, we explored the activation of AhR pathway by Sema4D in EL-4 cells with anti-CD3/CD28 antibodies treatment. Results showed that Sema4D enhanced the protein and mRNA expression of CYP1A1, and the enzymatic activity of CYP1A1 under Th17 or Treg cell polarization conditions (Figures 5A–C), indicating that Sema4D might mediate AhR pathway activation in lymphocyts. Subsequently, we performed a blocking assay using siAhR plasmid and AhR antagonist CH233191. AhR antagonist or siAhR were shown to decreased Sema4D-induced CYP1A1 enzymatic activity, the mRNA and protein expression of CYP1A1 (Figures 5D–F). These results suggested that Sema4D-mediated effects on CYP1A1 were dependent on AhR. To further confirm that the Sema4D-mediated CYP1A1 activity was attributed to the increase of XRE (AhR-dependent reporter gene) reporter activity, we transfected EL-4 cells with an XRE-driven luciferase reporter gene and siAhR plasmid. Sema4D was shown to promote the reporter gene expression through AhR activation, and siAhR plasmid almost completely reversed Sema4D-promoted the reporter gene expression (Figure 5G). These findings revealed that Sema4D could significantly promote AhR binding to a specific XRE sequence and that AhR was a key factor in the Sema4D-mediated Th17 cell and Treg cell differentiation.

### Sema4D-CD72 Interaction Is Required for Cytokine Production and AhR Activation

As the Th17-related cytokines IL-17 and IL-22 play a critical role in the pathogenesis of AS, we evaluated the effect of Sema4D on the cytokine production profile of T cells. Culture supernatants were collected after CD4 + T cells were incubated with or without Sema4D. IL-17 and IL-22 cytokine levels were markedly elevated after Sema4D stimulation (Figures 2F,G).

**FIGURE 6:**
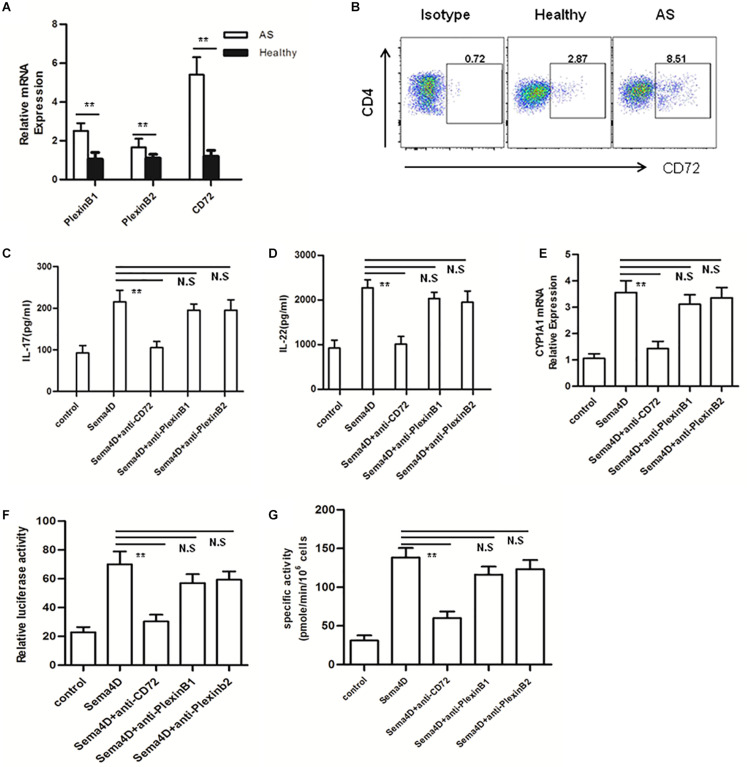
Blocking CD72 ligand abrogates Sema4D-induced cytokine production and AhR activation. **(A)** Expression of mRNA for Sema4D receptors (plexin B1, plexin B2 and CD72) in peripheral blood CD4 + cells from AS patients and healthy controls. Results shown are from 20 patients with AS and 15 healthy controls. **(B)** CD72 expression on CD4 + cells from 20 patients with AS and 15 healthy controls was analyzed by flow cytometry. **(C,D)** IL-17 and IL-22 was quantified in the supernatant of CD4 + T cells from Healthy (*n* = 15) and AS patients (*n* = 20) treated with anti-plexin B1, plexin B2 or CD72 antibody, respectively 10 μg/ml, in the presence of soluble Sema4D (10 ng/ml) by ELISA. **(E)** Expression of mRNA for CYP1A1 in peripheral blood CD4 + cells from 20 patients with AS and 15 healthy controls treated with anti-plexin B1 (10 μg/ml), plexin B2 (10 μg/ml) or CD72 (10 μg/ml) antibody in the presence of soluble Sema4D (10 ng/ml). **(F,G)** EL-4 cells were treated with anti-CD3/CD28, anti-plexin B1 (10 μg/ml), plexin B2 (10 μg/ml) or CD72 (10 μg/ml) antibody in the presence of soluble Sema4D (10 ng/ml) for indicated times. After 48 h, the activity of XRE-driven luciferase reporter gene and enzymatic activity of CYP1A1 were detected by using luciferase report gene and EROD assay, respectively. The data were presented as means ± SEM. The results are representative of three independent experiments. ** = *p* < 0.01; N.S = no significant.

The effects of Sema4D are believed to be regulated via three receptors: Plexin B1, Plexin B2, and CD72 (33). The effects of Sema4D are believed to be regulated via three receptors:PlexinB1,PlexinB2, and CD72 (32–34). To determine which receptor is involved in the stimulatory effect of Sema4D observed in CD4 + T cells, we first used qRT-PCR to evaluate the transcription of Plexin B1, Plexin B2, and CD72 in CD4 + T cells derived from patients with AS and healthy subjects. After Sema4D stimulation, the mRNA levels of CD72 were evidently upregulated compared to those of Plexin B2 and Plexin B1 (Figure 6A). Then, we used flow cytometry to measure the cell surface expression of the CD72 protein on CD4 + T cells derived from AS patients and found that after Sema4D stimulation, CD72 expression was increased on CD4 + T cells derived from both AS patients and healthy controls (Figure 6B).

To further address the role of CD72 in Sema4D-mediated cytokine production and AhR activation, blocking assays were conducted with anti-Plexin B1, anti-Plexin B2 or anti-CD72 antibodies. CD4 + T cells were stimulated with Sema4D or with anti-plexin B1, anti-plexin B2 or anti-CD72 antibodies, and cytokine production was assessed. Sema4D-induced cytokine secretion was significantly suppressed in CD4 + T cells treated with the anti-CD72 antibody compared with control cells in the presence of either the anti-plexin B1 or anti-plexin B2 antibody (Figures 6C,D). Furthermore, incubation with the anti-CD72 antibody markedly attenuated Sema4D-induced XRE-dependent luciferase reporter gene expression and the enzymatic activity and mRNA expression of CYP1A1 (Figures 6E–G). These findings suggested that Sema4D may induce cytokine secretion and AhR activation in CD4 + T cells via interactions with the CD72 receptor. These observations further reinforced the possibility that CD72 may be the major receptor controlling the effect of Sema4D signaling on the proliferation and differentiation of CD4 + T cells in AS patients.

## Discussion

Immunological inflammation has been recognized as the critical factor in AS pathogenesis, and disrupted immune homeostasis is closely related to the occurrence of immune pathologies (34). Previous work demonstrated that skewing of the Th17/Treg balance toward Th17 polarization plays a pivotal role in AS (35). Recent work has shown that Sema4D is expressed in immune cells and osteoclasts from RA patients and plays a critical role in RA pathogenesis (19, 29). Consistent with these findings, we found in this study that the serum Sema4D concentration was significantly increased in the peripheral blood of AS patients. These data suggested that Sema4D might be involved in the pathogenesis of AS. Although recent reports have shown that serum Sema4D concentrations are elevated in other disease states and animal models, the role of Sema4D in AS patients has not yet been elucidated. Our results highlight the pathological significance of Sema4D in AS pathogenesis, suggesting basic research and clinical implications.

Sema4D is abundantly expressed on T cells and weakly on B cells. and can be induced to shedding from the cell surface by matrix metalloproteases to become soluble Sema4D (sSema4D). Both membrane-bound Sema4D (m Sema4D) and sSema4D have important immune regulatory functions that promote immune cell activation and responses (36, 37). Sema4D exerts essential functions in T cell priming and activation. In line with these findings, Our data verified the expression and role of sSema4D in regulating immune responses in the pathogenesis of AS.

Previous studies have indicated that soluble Sema4D levels are elevated in RA patients and that high soluble Sema4D levels are related to clinical and biological markers of RA (29). Therefore, we investigated the role of Sema4D in the pathogenesis of AS. In the current study, we confirmed that the serum concentration of Sema4D was significantly increased in patients with AS and positively correlated with markers of AS disease activity, such as the CRP level and BASDAI score. In addition, we found that the change in the soluble Sema4D concentration before and after treatment of AS with a TNF-α blocker and the decrease in the concentration of soluble Sema4D in AS patients after successful treatment were positively correlated with lower clinical disease activity. Our data suggest that Sema4D is an additional clinical disease marker for the evaluation of AS activation status. Recent reports have shown that the expression of Sema4D is elevated in osteoclasts and induces bone resorption by activating osteoclasts (38). In the current study, we measured the serum levels of several bone markers to explore whether bone formation or bone resorption are affected by Sema4D in AS patients. the levels of the resorption indexes CTX and TRAP 5b, which indicate osteoclast activity, were significantly elevated and correlated with the serum Sema4D concentration in AS patients. However, only a weak correlation was observed between the serum Sema4D concentration and the levels of the bone formation markers osteocalcin and BAP. Collectively, these results suggest that Sema4D enhances bone resorption in AS patients. Further work will be needed to ascertain the exact role of Sema4D in bone loss in AS.

Immune inflammation in AS is classically characterized by activation of inflammatory pathways in both the innate and adaptive immune responses. The manifestations of this immune inflammation are driven by T cells, especially CD4 + T cells (35). Significant increases in Th17 cells and significant decreases in Treg cells in the peripheral blood of AS patients were found to be responsible for the pathogenesis of AS (39). As recent reports indicated that Sema4D is expressed mainly in T cells (40), we assessed the cell surface expression of Sema4D by flow cytometry and the mRNA expression of Sema4D by qRT-PCR. Intriguingly, in CD4 + T cells from AS patients, the cell surface expression of Sema4D was significantly reduced, whereas the mRNA expression of Sema4D was elevated. This relative reduction in the cell surface level of Sema4D is reported to be attributable to shedding of Sema4D from the surface of CD4 + T cells. Collectively, our results indicated that the reduction in cell surface membrane-bound Sema4D is crucially implicated in the development of AS and prompted us to investigate the effect of Sema4D on CD4 + T cell proliferation and differentiation.

Consistent with previous data (39), our data confirmed that the proportion of Th17 cells is significantly increased in the PBMCs of AS patients. We investigated the function of Sema4D during CD4 + T cell proliferation. After stimulation with Sema4D, CD4 + T cells exhibited significant proliferation in a culture system of purified CD4 + T cells from AS patients. However, this proliferation was significantly suppressed by the addition of soluble anti-Sema4D. Similar to FICZ, Sema4D remarkably boosted both the Th17 cell frequency and cytokine secretion in AS patients, and the level of secreted IL-17 and the mRNA expression levels of IL-17 and RORγt in CD4 + T cells from AS patients were markedly elevated after treatment with soluble sema4D treatment. In addition, Tregs are essential for suppressing excessive autoimmune responses, thereby maintaining immune homeostasis. Our findings indicated that Sema4D can suppress Foxp3 and IL-10 expression, suggesting a role of Sema4D in the functional impairment of Treg cells in AS patients. These data revealed that Sema4D might mediate the pathogenesis of AS by inducing T cell proliferation, regulating Th17 and Treg cell differentiation and reinforcing Th17 cell function. In addition, it is also interestingly that increased percentages of CD4 + CD25-Foxp3 + cells in AS after Sema4D stimulation. However, Yang and colleagues reported that the phenotype and functional activity of Tregs were scarce on CD4 + CD25-Foxp3 + in lupus patients, that not all CD4 + Foxp3 + T cells have protective suppressive function. Also, it has been reported that CD4 + CD25-Foxp3 + T cells functionally promote the proliferation and differentiation of CD4 + T cells into Th17 cells might sustain chronic inflammation (41–43). Therefore, further study will be needed to explore the exact role of Sema4D-mediated CD4 + CD25-Foxp3 + cells elevation in AS. Taken together, the current findings further illuminated the immunoregulatory mechanism of Sema4D that causes the Th17/Treg imbalance.

Recently, AhR has attracted researchers’ attention because of its widespread expression in immune cells, such as certain subtypes of T cells, Th17 and Treg cells. AhR is involved in critical immunoregulatory functions (44). In many autoimmune diseases, AhR activation is the crucial factor regulating the differentiation of Th17 and Treg cells (45). Thus, we speculated that Sema4D regulates Th17 and Treg cell differentiation in a manner dependent on the activation of AhR to accelerate AS development. Here, we found that Sema4D enhanced the expression of AhR and CYP1A1 (the AhR target gene) in AS patients. Notably, pretreatment of Sema4D-exposed cells with an AhR antagonist or knockdown of AhR significantly decreased the expression of Cyp1A1 and suppressed the regulatory effect of Sema4D on Th17 and Treg differentiation. Moreover, Sema4D potentiated CYP1A1 enzymatic activity via the AhR pathway. Here, we conducted further detailed analysis and verified that Sema4D-induced CYP1A1 activity was attributable to increased expression of an AhR-dependent reporter gene. These results further corroborated the hypothesis that Sema4D can induce AhR pathway activation to modulate Th17 and Treg differentiation in AS patients.

Sema4D has been reported to function as a ligand and to interact with three different receptors: Plexin-B1, Plexin-B2, and CD72 (46). To determine which receptor is involved in the Sema4D-induced activation of AhR, we first measured the expression levels of PlexinB1, PlexinB2, and CD72 in activated T cells and found that CD72 is the main Sema4D receptor in CD4 + T cells from AS patients. It should be noted that treatment with an anti-CD72 antibody significantly suppressed CYP1A1 mRNA expression and XRE reporter activity in CD4 + T cells in the presence of Sema4D. Moreover, cytokine secretion was suppressed. These data implied that the promotive effects of Sema4D on AhR activation and cytokine production are dependent on its interaction with the CD72 receptor.

## Conclusion

This work is the first to report the role of Sema4D in AS patients. Our results showed that the serum concentrations of secreted Sema4D were significantly higher in AS patients than in individuals without AS and that Sema4D is a potential biomarker for AS disease. Furthermore, our studies aimed to reveal the mechanism by which increased Sema4D levels play a role in regulating the immune response in AS patients. Therefore, further studies should also address the questions of whether sSema4D represents a useful biomarker for evaluating the immune response in patients with AS and whether it could serve as a potential therapeutic target for AS treatment.

## Data Availability Statement

The datasets presented in this study can be found in online repositories. The names of the repository/repositories and accession number(s) can be found in the article/supplementary material.

## Ethics Statement

The participants gave their written informed consent and the Regional Ethics Committee at Nanjing Medical University approved the study.

## Author Contributions

JX conceived and designed the study, analyzed the data, and planned the experiment. JX, ZW, and WW performed *in vitro* experiments and participated in its design and coordination, and drafted the manuscript. All authors read and approved the final manuscript.

## Conflict of Interest

The authors declare that the research was conducted in the absence of any commercial or financial relationships that could be construed as a potential conflict of interest.
